# Coiled-coil domains are sufficient to drive liquid-liquid phase separation of proteins in molecular models

**DOI:** 10.1101/2023.05.31.543124

**Published:** 2023-06-03

**Authors:** Dominique A. Ramirez, Loren E. Hough, Michael R. Shirts

**Affiliations:** 1 Department of Biochemistry, University of Colorado, Boulder CO, 80309; 2 Department of Physics and BioFrontiers Institute, University of Colorado, Boulder CO, 80309; 3 Department of Chemical and Biological Engineering, University of Colorado, Boulder CO, 80309

## Abstract

Liquid-liquid phase separation (LLPS) is thought to be a main driving force in the formation of membraneless organelles. Examples of such organelles include the centrosome, central spindle, and stress granules. Recently, it has been shown that coiled-coil (CC) proteins might be capable of LLPS, such as the centrosomal proteins pericentrin, spd-5, and centrosomin. CC domains have physical features that could make them the drivers of LLPS, but it is unknown if they play a direct role in the process. We show, using coarse-grained models, that the physical features of CC domains are sufficient to drive LLPS of proteins. We developed a coarse-grained simulation framework for investigating the LLPS propensity of CC proteins, in which interactions which support LLPS arise solely from CC domains. The framework is specifically designed to investigate how the number of CC domains, as well as multimerization state of CC domains, can affect LLPS. We show that small proteins with as few as two CC domains can phase separate. Increasing the number of CC domains up to four per protein can somewhat increase LLPS propensity. We demonstrate that trimer-forming and tetramer-forming CC domains have a dramatically higher LLPS propensity than dimer-forming coils, which shows that multimerization state has a greater effect on LLPS than the number of CC domains per protein. These data support the hypothesis of CC domains as drivers of protein LLPS, and has implications in future studies to identify the LLPS-driving regions of centrosomal and central spindle proteins.

## INTRODUCTION

Liquid-liquid phase separation (LLPS) is important for the formation of biomolecular condensates. LLPS occurs in cells when macromolecules interact favorably with each other, demix from bulk cytosol, and form a concentrated coexisting phase. LLPS provides a mechanism for cells to localize protein and chemical function (1) without the need for membrane enclosure into aptly named membraneless organelles (1–4). Examples of membraneless organelles that are thought to form via LLPS include P-bodies, nucleoli, stress granules (5, 6), centrosomes (7–9), and the central spindle (10). The formation of condensates *in vitro* is well supported but the role of biologically relevant LLPS, specifically in the biogenesis of membraneless organelles, is actively debated.

There is recent evidence that coiled-coil (CC) proteins have the propensity for LLPS. The formation of the centrosome and central spindle in particular seems to rely on the LLPS of CC proteins. Examples of phase-separating centrosomal proteins include: spd-5 (11), centrosomin (12), and pericentrin (13, 14). MAP65 and its mammalian homolog PRC1, both of which help form the central spindle, can undergo LLPS as well (10). Experiments with pericentrin and centrosomin suggest that CC domains are important for these proteins’ phase separation propensity (12, 14). There are other CC proteins that LLPS, such as the Golgi structural protein GM130 (15), the measles phospho-protein (16), transcription factors FLL2 (17) and TAZ (18), the endocytic trafficking protein Ede1 (19), a protein encoded by retrotransposable element LINE-1 ORF1 (20), and the RNA-binding protein Whi3 (21). Truncation studies of TAZ and Ede1 that lack their CC domains show that these mutants lose the ability to LLPS, compared to wild-type (18, 19). These studies support a narrative that CC domains are involved in LLPS, but it is unknown how they might be contributing and if they themselves are sufficient to drive the process. We are motivated by these examples of phase-separating CC proteins and in this paper we answer the question: Could coiled-coil domains themselves be sufficient to drive the LLPS of proteins?

LLPS is typically driven by multivalency (22–24), which is the ability of molecules to form many attractive interactions with other, similar molecules. A useful conceptual framework for LLPS driven by multivalent interactions is the stickers-and-spacers model of associative polymers (22, 25). In this framework, stickers are specific macromolecular components (e.g. residues, domains, or globular patches on proteins) which interact associatively, whereas spacers are the remainder of the macromolecule that tether the stickers together. This framework applies equally well to intrinsically disordered proteins (IDPs) with stickers being individual residues, as it does for proteins where folded domains are stickers and the spacers are flexible linkers tethering the domains together. In these cases, multivalency increases as the number of stickers in a protein increases. There are a few examples which show the effect of folded-domain-stickers on protein LLPS, such as the synthetic SH3-PRM system introduced by Li et al. (26) and ubiquitin shuttle proteins p62 and UBQLN2 (27, 28). In the rest of the paper, we use the term *polymeric multivalency* to refer to the number of stickers in a single protein chain.

Multimerization of individual stickers is a plausible, but not well explored, multivalency mechanism in proteins. Protein domain multimers that are larger than a dimer, in particular, have mostly been ignored in the discussion of multivalency in proteins. This is likely because sticker interactions are often assumed to be 1:1, meaning the entirety of one sticker interacts only with the entirety of one other sticker. This is an assumption that comes from the simplifications necessary in polymer theory models e.g. theory of associative polymers (22, 25). Higher orders of multimers likely occurs naturally between residue-level stickers, however. Charged (29) and polar amino acids can engage in multiple interactions simultaneously, for example in the coordination of metal ions (30, 31). Multivalent interactions for residue-level stickers can be an important factor in protein LLPS (32, 33). Multimerization between entire protein domains can also occur naturally to increase LLPS propensity (28), and this requires treating protein domains entirely as individual stickers. Work by Carter et al. (34) showed that mutants of TDP-43 containing a tetramerization domain have higher LLPS propensity than wild-type TDP-43 with its dimerization domain. Some examples of LLPS affected by multimerization have been reported (summarized by Mohanty et al. (28)), but beyond these few the role that multimerization plays in affecting multivalency has not been well explored. In this paper we use the term *multimeric multivalency* to refer to the number of partners that an individual sticker is capable of interacting with at once. We use the familiar terms dimer, trimer, tetramer, etc., to describe specific varieties of multimers.

CC proteins might utilize both polymeric and multimeric multivalency mechanisms to drive LLPS through their CC domains. If we apply the stickers-and-spacers framework to CC proteins and treat the CC domains as the stickers, we see that these proteins harness both polymeric and multimeric multivalency. CC proteins can show varying degrees of polymeric multivalency dependent on the number of CC domains in the protein. CC domains also have intrinsic multimeric multivalency because of their ability to form dimers (35), trimers, tetramers, and larger multimers (36). Proteins containing many CC domains could realize LLPS through a combination of polymeric and multimeric multivalence mechanisms; the extent to which this may happen is not understood.

**Figure 1:**
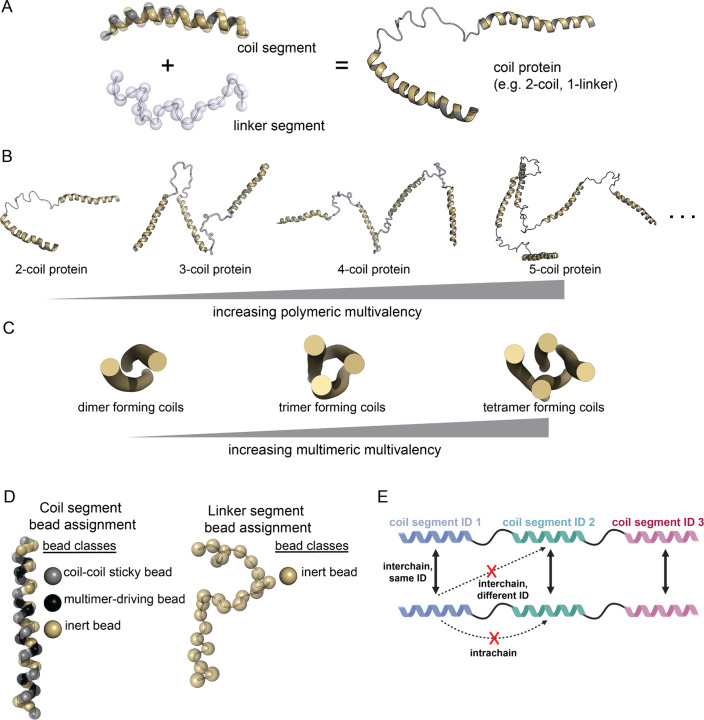
The CC LLPS framework can be used to make CC proteins with varying polymeric and multimeric multivalency. (A) Coil segments and linker segments can be combined in any number to make a coil protein, e.g. a 2-coil-1-linker protein. Haloed spheres represent the CG beads, and are removed in later visualizations for clarity. Helices are the coil segments and the silver portions are the coil-coil sticky beads. White tubes are the linker segments. (B) The polymeric multivalency of a CC protein can be modified by changing the number of coil segments in the protein. Only two through five coil segments are shown, but there could be an arbitrarily large number of coil segments per protein (represented by ellipsis). (C) The multimeric multivalency of a protein can be modified by changing the type of multimer that a coil segment is favored to form. (D) Diagram showing how the different bead classes are organized within each of the segments. (E) Schematic representation of the *maximally specific interaction scheme* used in this study, where only interchain interactions between the same type of coil segment is permitted, indicated by a solid black line. Schematic made using BioRender. All molecular visualizations were produced using open-source PyMOL v.2.5.0.

We hypothesize that CC domains are sufficient to drive the LLPS of proteins and that this phenomena can be tuned by both their polymeric and multimeric multivalency. We have generated a novel coarse-grained framework to test our hypothesis by molecular simulation (Fig. 1). We use this framework to build CC proteins where CC domains are tethered to other CC domains with disordered, nonattractive linker regions (Fig. 1A). This framework allows us to specify the polymeric multivalency of CC proteins by changing how many CC domains are in a given protein chain (Fig. 1B), as well as the multimeric multivalency of each CC domain (Fig. 1C). For the purposes of this paper, we restrict our attention to dimer-, trimer-, and tetramer-sized multimers. We use this framework to focus on general physical properties of phase separation of CC proteins when treated as associative polymers. We show that CC domains can be sufficient to drive LLPS of proteins when only interchain multimers are permitted, and that multimeric multivalency greatly influences the LLPS propensity of proteins.

## METHODS

### Software

All molecular dynamics (MD) simulations were performed using unmodified GROMACS (37) version 2022.1. GROMACS was compiled with GCC version 11.2.0 and Open MPI version 4.1.1 for parallel processing on the Alpine super computer, and with GCC version 10.2.0 and Open MPI version 4.0.5 for Bridges-2. Non-MPI enabled versions of GROMACS 2022.1 were used for small scale simulations, and some data analysis and processing. These versions were compiled on a Ubuntu machine (22.04.2 LTS) with GCC 11.2.0 and MacOS 12.6 with AppleClang 14.0.0. Additional analysis code was written in Python 3.9.

### Code availability

Files from this study, including molecular dynamics parameter files, relevant topologies, analysis scripts, etc. are available on the GitHub repository associated with this manuscript: https://github.com/dora1300/cc_llps_framework.

### Coarse-grained framework to study coiled-coil driven LLPS

We developed a bespoke, coarse-grained (CG) molecular dynamics framework to study CC LLPS, inspired by other types of CG models of CC proteins (38, 39). Computational methods have been critical in the understanding of protein LLPS (23), but prior to this study there were no tools specifically designed for studying CC-driven LLPS. We designed our framework to be easy to use and readily modifiable so it can represent a variety of different CC proteins of arbitrary size (Fig. 1B, with coils of any desired multimerization state (Fig. 1C), and of defined specificity between coil segments (Fig. 1E).

We define CC proteins as linear combinations of *coil segments* and *linker segments* (Fig. 1A), which is inspired by the organization of centrosomal proteins (40). Coil segments are regions of the protein which represent CC domains, and linker segments are the tethers between coil segments. We specifically chose this language to avoid confusion with terms like ‘domains’ from other fields. This organization allows us to treat CC proteins as associative polymers, where each coil segment acts as a single sticker and each linker segment as a spacer. We implemented the framework with the following objectives and simplifying assumptions in mind: (a) coil segments are helical, and linkers are disordered, (b) protein–protein interactions only occur through coil segments, (c) coil segments retain a heptad-repeat like CG bead organization so that coils interact in a similar geometry to real CC domains, and (d) linkers interact equally between solvent and protein components.

Our framework uses C-*α* coarse-graining, which represents every amino acid as a single CG bead. These beads are centered at the same coordinates as the C-*α* carbons of amino acids in a corresponding atomistic representation of a protein. The C-*α* CG procedure maps traditional *ϕ*,*ψ* angles into coarse-grained space (41). The corresponding CG pseudo-bonds and pseudo-torsions allow us to reproduce correct secondary structure geometries in the CG structures. A CG representation thus has significantly fewer beads than an atomistic representation (on average, 19 atoms are replaced by one bead), which greatly increases computational efficiency in molecular simulation, as well as increasing the time step allowed by increasing the masses of the smallest particle. Additional details about the framework parameters are provided in supplemental methods.

### Common molecular dynamics parameters

We used a consistent set of MD run parameters throughout the study, and deviations from these parameters are noted in the respective methods. Energy minimization was performed using steepest descent to a force tolerance of *<* 50 kJ/mol/nm and a step size of 2 pm. Equilibration and production simulations were done with Langevin dynamics using the sd integrator with a friction coefficient of 0.2 ps^−1^. The center of mass translational velocity was removed every 10 steps. Lennard-Jones potentials were shifted and cut-off at 1.1 nm, consistent with other coarse-grained force fields (42). The verlet-buffer-tolerance, a GROMACS-specific setting for Verlet list updates, was set to 1 × 10^−7^. Setting this parameter correctly is important for reproducibility because using default larger numbers for verlet-buffer-tolerance results in simulation instability, due apparently to density inhomogeneity in phase-separated simulation boxes. Random seeds for each simulation were generated pseudo-randomly by GROMACS. Periodic boundary conditions were applied in all three dimensions. We used a single box size for slab simulations to maintain consistent configurational entropy between experiments. The final box size for slab simulations is 25×25×150 nm, which is similar to previous studies (5).

### Temperature replica exchange MD to assess dynamics of coarse-grained CC dimer

We verified that simulated CG dimers reproduce similar structure to atomistic simulations of a CC dimer. Thomas et al. (43) performed 100 ns atomistic temperature replica-exchange (REMD) of two, 32-amino acid long coiled-coils starting in a dimer configuration and published the backbone RMSD of each replica. We reconstructed a mean RMSD distribution of their replicas to serve as our reference for a stable, CC dimer (Fig. S2A). We performed simulations of a CG CC dimer in our framework and compared our backbone RMSD distribution to the reference. Specific details are provided in supplemental methods. Figure S2B–C shows the backbone RMSD distributions across different coil-coil interaction strengths, at 293 (Fig. S2B) and 310 (Fig. S2C) K. We generated Gaussian kernel density estimates for all of the RMSD distributions and determined the similarity between our CG RMSD data to the atomistic reference distribution by calculating the Kullback-Leibler (KL) divergence (Table S3). The lowest KL divergence, at both temperatures, was from the 5.5 kJ/mol simulations (298 K, 0.90 nats; 310 K, 1.01 nats). Thus, 5.5 kJ/mol is chosen as the optimal coil-coil sticky interaction strength (for dimer-forming coils) for our framework. This validation demonstrates that our CG framework can reproduce a similar structure and equilibrium folding propensity as an atomistic coiled-coil dimer.

### Single molecule MD of intrinsically disordered proteins to parameterize linker segments

We fine tuned the linker segments by systematically weakening the magnitude of the linker structural parameter force constants starting from the coil segment values, and determined the optimal force constants by comparing simulated *R*_*g*_ of all 23 IDPs against the experimentally determined *R*_*g*_ (details in supplemental methods). The interaction parameters of the inert beads was left unaltered in all cases. Figure S3 shows a parity plot comparing simulated to experimental *R*_*g*_ for the 23 tested proteins, with structural parameter force constants 100× weaker than coil segment (i.e. helix forming) parameters. This level of weakening produced the best correlation between simulated and experimental data. The Pearson correlation coefficient between simulated and experimental data is 0.753. The slope of the simulated versus experimental data is 0.814 and is qualitatively close to the parity line. We judge that this qualitative agreement to real IDPs is sufficient for this study, even in the absence of amino acid-specific chemical information and attractive interactions between the inert linker beads.

### MD to assess multimerization capacity of coil segments

We confirmed that we have moderate control over the multimerization of coil segments in the CC LLPS framework. We verified this by simulating several copies of a 3-coil-2-linker protein with each of the multimer-driving bead types and quantified the multimer species present in the simulations (details provided in supplemental methods). Simulations with the dimer multimerization beads (Fig. S4A) show that dimers are the dominant multimer present at about 60% composition over simulation time. Simulations with the trimer multimerization beads (Fig. S4B) produce a mixture of multimers with the dominant multimer species being the trimer at 46% of composition averaged over time. Tetramer multimerization beads also produce a mixture of multimers (Fig. S4C) with the trimer and tetramer multimer species comprising 38% and 26% of composition over time, respectively, at a coil sticky interaction strength of 4 kJ/mol. Attempts to shift the multimer population towards the tetramer species by changing the coil interaction strength either ablated multimer formation (3 kJ/mol) or resulted in pentamers appearing in the simulation (5 kJ/mol), suggesting that 4 kJ/mol is near optimal strength for tetramer-forming beads (Fig. S4C).

It was necessary to lower the coil sticky interaction parameter (i.e. the effective pair interaction strength parameter) for the trimer- and tetramer-forming models relative to the dimer-forming model to prevent aberrantly large multimers from forming. Increasing the strength for all types of multimer-driving bead interactions resulted in multimers larger than desired. We determined the current implementation of multimer-driving beads to be sufficient because even though we did not achieve ideal multimer specificity, none of the beads produced aberrantly large multimers. The lack of full specificity in the trimer- and tetramer-forming coils might be a consequence of using a C-*α* coarse-graining approach, but this remains to be tested.

### Slab simulations for phase coexistence

We used the slab simulation (5, 44, 45) protocol to directly simulate phase coexistence of coiled-coil protein models. This procedure involves the following steps: generating configurations of each protein of interest, randomly placing copies of those configurations in a simulation box, compressing the box in one dimension in the NPT ensemble to generate the slab, expanding the box in the same compressed dimension, equilibrating the slab to the desired temperature in the NVT ensemble, and, finally, production simulation in the NVT ensemble.

#### Single molecule simulations to generate starting configurations of CC proteins

We generated proteins of interest using the PeptideBuilder strategy (described in supplemental methods) and then used MD to equilibrate the structure of each protein and prepare it for slab simulation. A single copy of each protein is placed into a simulation box large enough to accommodate the initial structure. Single molecules were energy minimized and then equilibrated in the NVT ensemble for 500 ps with a time step of 25 fs. NVT equilibrations were used so that the linker segments could relax out of the initial helical configuration and closer to the defined equilibrium angles. At this point, for each protein structure we generated a unique single molecule simulation for every temperature that would be used in the forthcoming slab simulations, i.e. 253, 273, 293, and 313 K. Production MD simulations in the NVT ensemble were performed on all equilibrated structures for 5 *μ*s with a time step of 25 fs at their corresponding temperatures. Only 1 replicate simulation was used for each single molecule simulation at each temperature.

#### Selecting single molecule configurations for slab simulation

We begin the slab protocol by randomly packing copies of our proteins of interest in a simulation box. The single molecule simulations served as the pool of configurations from which we pack the starting box. We standardized the total number of coil segments in a simulation box to 450 coils for all protein models. Simulations with 4-coils per protein had a total of 448 coils in each box. We mitigated configurational bias by randomly choosing five configurations from the equilibrated portion of the single molecule simulations (equilibrated state began by 100 ns), using random numbers generated from Random.org. Each of the 5 configurations from each protein was copied so that the total coil density in the simulation would reach the desired total coil density (450). We prepared three replicate simulations at every desired temperature, with different random numbers for each replicate, using only the corresponding single molecule trajectories to provide the structures. packmol v20.11.1 (46) was used to pack all the initial slab boxes, with a tolerance of 1.0 nm between every protein copy, into a box size of 25×25×100 nm to ensure all copies would fit inside. We generated initial slab simulation boxes for every protein of interest, at every desired temperature, in triplicate.

#### Slab simulations

Packed boxes were energy minimized and then equilibrated in the NPT ensemble for 200 ns with a time step of 20 fs at 150 K. The goal of this step is to create the actual protein slab to assess phase coexistence. Semi-isotropic pressure coupling was applied only in the z-dimension of the box. The Parrinello-Rahman barostat was used with a reference pressure of 1 bar, compressibility of 3 × 10^−4^ bar^−1^, and a time constant of 5 ps for the coupling. Simulation boxes are compressed to approximately 10–20% of the starting z-axis length at the end of NPT equilibration. Proteins were “unwrapped” prior to expanding the box to prevent aberrant expansion of individual proteins. The protein slab was kept centered in the simulation box during expansion, at which point the z-dimension was increased to a final length of 150 nm. Slabs in expanded boxes were then equilibrated in the NVT ensemble for 200 ns with a time step of 20 fs, at the desired temperatures. We then performed production MD simulations for each temperature-equilibrated slab for a total of 20 *μ*s with a time step of 25 fs, with the GROMACS parameter rdd set to 1.6 nm (important for domain decomposition). We found that long simulation times for our proteins are necessary when compared to other studies with the slab method (5, 45, 47), to ensure that our systems reach equilibrium. We do not expect this difference to be due to protein length, because the number of beads in our proteins and systems are similar to sizes tested by Dignon et al. (5). It is not clear what features of our framework require longer slab simulation times. Equilibrium was assessed by monitoring the density of both the center of the box, and the opposite ends of the box combined. We considered the simulation/slab to be equilibrated when the density of both these regions was stable. Only the equilibrated portions of trajectories were used for analysis. Almost half of all simulations equilibrated within 2.5–5 *μ*s, but some simulations, particularly for the 4-coil containing proteins and tetramer-forming coils, took 10 *μ*s to equilibrate.

### Density profile analysis and calculation of binodals

We performed density analysis of each equilibrated trajectory using the gmx_density module in GROMACS. Density (reported as number density / nm^3^) was calculated in 2 nm slices along the z-axis of the simulation box, and the average density in each 2 nm slice for a trajectory is output. Density profiles (which are reported along the z-axis) for a given protein at a given temperature were averaged across three replicate simulations and are plotted as mean (solid line) ± standard deviation (shaded region). These data are used in determining LLPS, in combination with molecular cluster analysis (described below). We also used the density output to calculate binodals. For a single trajectory, number densities in the center of a slab corresponding to 70–80 nm in the z-dimension was averaged to describe the denser region’s density. Number densities at the edges of the box not corresponding to a slab, ranging from 1–35 and 115–150 nm in the z-dimension, were averaged to represent the dilute region. Dense and dilute regions across triplicate simulations were averaged and plotted along with standard deviation in binodals.

### Molecular cluster analysis

Molecular cluster size distributions were determined from every 10 ns of equilibrated trajectories using the gmx_clustsize module in GROMACS. Cluster sizes for whole molecules were calculated using a distance cut-off of 0.9 nm between interacting pairs. The output cluster size populations were normalized to probabilities and averaged for each temperature replicate for each protein of interest, and are reported as mean ± standard deviation over three replicate simulations. Molecular cluster analysis is used to help determine LLPS in slab simulations, in combination with density profile analysis. Both a molecular cluster containing nearly all of the proteins in a simulation and the simultaneous presence of a density transition in the *z*-direction are markers of LLPS.

## RESULTS

Dimer-forming coil segments can drive LLPS of CC proteins

We hypothesized that CC proteins would readily form LLPS droplets in simulation due to physical features of CC domains that could enable both polymeric and multimeric multivalency mechanisms. We developed a custom simulation framework to study the phase separation of CC proteins, and observed LLPS over a range of polymer multivalency, multimer multivalency, and temperatures.

We first investigated whether a relatively small number of coil segments was sufficient for LLPS. We tested three coarse-grained CC proteins — 2-coil-1-linker, 3-coil-2-linker, and 4-coil-3-linker (Fig. 1B, first three proteins shown), all with dimer-forming coils — for LLPS propensity using the slab method. A LLPS droplet is determined quantitatively from density profile and molecular cluster analyses (see Methods) and exists when there is both a sharp density transition and the presence of a molecular cluster whose size is close to the number of proteins in the simulation. Averaged density profiles and molecular cluster size distributions for all simulated proteins are reported in Figure S5.

**Figure 2:**
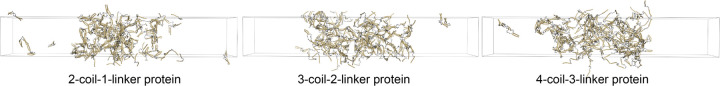
Coil segments can drive LLPS of CG proteins. Snapshots (at 20 *μ*s) from slab simulations at 253 K of a 2-coil-1-linker protein (left), 3-coil-2-linker protein (middle), and 4-coil-3-linker protein (right), all with dimer-forming coils. Proteins are made whole for visualization, but would wrap through the box boundaries during actual simulation. Visualizations produced using open-source PyMOL v.2.5.0.

**Figure 3:**
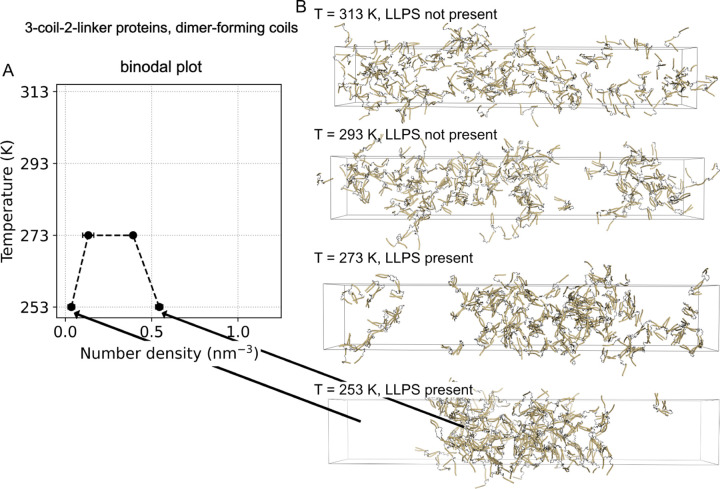
LLPS behavior varies with temperature for dimer-forming coil proteins. (A) Binodal plot for a 3-coil-2-linker protein, which also appears in Fig. 5B. Data points on the left-hand side are for dilute regions, and right-hand side are for dense (droplet) regions. No points are shown at 293 K and above as no phase separation occurs at those temperatures. Data points are presented at mean (circles) ± standard deviation (bars) from three replicate simulations at each temperature. (B) Snapshots (at 20 *μ*s) from slab simulations from one replicate set for a 3-coil-2-linker protein with dimer-forming coils. Labels corresponding to qualitative assessments of LLPS accompany each snapshot. The snapshot at 253 K is the same as in Figure 2. Proteins are made whole for visualization, but would wrap through the box boundaries during actual simulation. Visualizations produced using open-source PyMOL v.2.5.0.

All three CC proteins formed LLPS at 253 K, the lowest of the temperatures that we simulated (reflected in representative snapshots in Fig. 2 and in phase diagrams in Fig. S8A). At higher temperatures (T ≥ 293 K), LLPS is not observed and the proteins are dilute throughout the system. The 3-coil and 4-coil proteins also formed LLPS droplets at 273 K, unlike the 2-coil protein. Increases in polymeric multivalency is known to increase LLPS propensity (2, 26, 48, 49), and our observations for dimer-forming coils are in line with these reports. Figure 3 shows a binodal plot for the 3-coil dimer-forming protein (Fig. 3A) along with a series of snapshots at each temperature corresponding to the points on the binodal (Fig. 3B). Snapshots of the 2-coil (Fig. S6A) and the 4-coil (Fig. S6B) dimer-forming proteins at each temperature are also presented. These combined data (Fig. 3 and S6) highlight that high temperatures destabilize LLPS droplets and result in dispersed proteins in simulation. These data demonstrate that dimer-forming coil segments are sufficient to drive LLPS of proteins, and that increasing polymeric multivalency has slightly increases LLPS propensity.

### Multimerization greatly affects LLPS propensity, whereas polymer multivalency only weakly affects LLPS propensity

**Figure 4:**
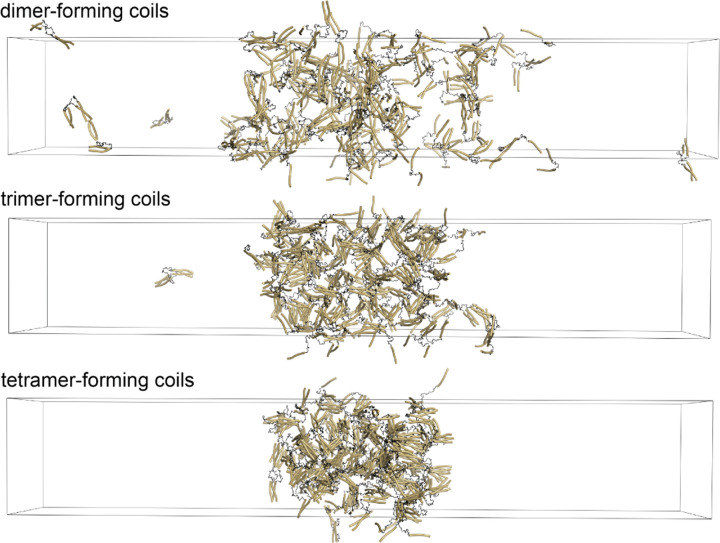
Multimer multivalency is associated with LLPS droplet compaction. Snapshots (at 20 *μ*s) from slab simulations for a 2-coil-1-linker protein. Each snapshot comes from one replicate at 253 K for proteins with dimer-forming (top, same as Fig. 2A), trimer-forming(middle), and tetramer-forming (bottom) coils. Proteins wrap through the box boundaries during simulation, but atoms are shifted into neighboring cells to keep proteins visually intact. Visualizations produced using open-source PyMOL v.2.5.0.

We varied the multimeric state of the three proteins (2-coil, 3-coil, and 4-coil proteins) to make trimer-forming and tetramer-forming versions. We ran slab simulations on these proteins and analyzed the trajectories for evidence of LLPS and inspection of density profiles and molecular cluster distributions show an increasing propensity for LLPS as multimer state increases (Fig. S5). We inspected the trajectories and observed a multimer-state dependent slab compaction, as demonstrated in Figure 4 for 2-coil proteins, whereby increasing the multimer state results in a more dense and compact LLPS droplet. This compaction behavior was observed for 3-coil and 4-coil proteins as well when inspecting representative snapshots (Fig. S7) and density profiles (Fig. S5A).

We next quantified the impact that polymeric and multimeric multivalency has on the LLPS propensity of our proteins. Our simulations show two results: (1) polymeric multivalency only slightly impacts LLPS of CC proteins, and (2) for a protein with fixed polymeric multivalence, increasing the multimer multivalency dramatically increases LLPS propensity. These results are shown by phase diagrams which compare the phase behavior for all tested proteins (Fig. S8). Increasing polymeric multivalency slightly increased LLPS for dimer-forming coils (Fig. S8A), but we did not see a similar trend for trimer-forming (Fig. S8B) or tetramer-forming (Fig. S8C) coils. We found that as the multimeric multivalency of the system increased, for a fixed polymer multivalence, the LLPS propensity was more robust, i.e. the apparent melting temperature for all proteins increased as the coil multimer-state increased (Fig. S8). The behavior of the 3-coil and 4-coil tetramer forming proteins might be different from each other above 313 K, but additional simulations closer to their melting points are needed.

**Figure 5:**
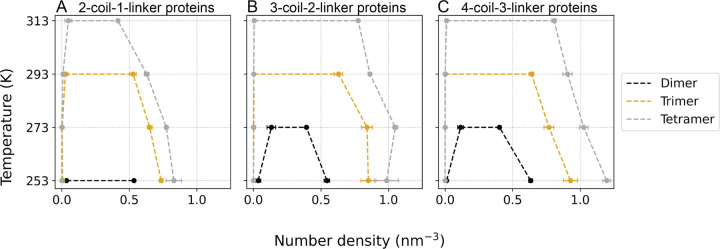
Multimeric multivalency greatly impacts LLPS propensity. Binodal plots for (A) 2-coil-1-linker proteins, (B) 3-coil-2-linker proteins, and (C) 4-coil-3-linker proteins, compare the effect that multimerization has on LLPS propensity. Data points on the left-hand side of plots are for dilute regions, and on the right-hand side are for dense regions. Data points are presented as mean (circles) ± standard deviation (bars) from three replicate simulations.

We constructed binodals from all of the simulations for a qualitative picture of LLPS for each of the proteins (see Methods). Figure 5 shows the binodals for the (A) 2-coil-1-linker, (B) 3-coil-2-linker, and (C) 4-coil-3-linker proteins, as coil-multimeric state is varied. Data points on the left side of each plot correspond to average densities from the dilute region of the simulation box, and data points on the right side of each plot correspond to average densities from the dense region. We omitted points at temperatures where the dense and dilute regions have overlapping number density, as this represents a melted regime and this data is not included in binodals. These data clearly show that increasing multimeric multivalency strongly increases LLPS propensity and melting temperature of protein droplets. The combination of Figure S8 and Figure 5 demonstrate that polymeric multivalence has a relatively small impact on the LLPS propensity of our CC proteins, whereas multimeric multivalence has a much greater influence.

## DISCUSSION

We report in this paper: (1) a novel CG framework for studying the phase separation of CC proteins (CC LLPS framework); (2) evidence that CC domains can be sole drivers of LLPS, at least in physically motivated coarse-grained protein models; and (3) evidence that multimerization of stickers has a strong influence on LLPS propensity. Our results show that increasing multimer multivalency has a stronger effect on LLPS propensity than increasing polymeric multivalency. It is notable that we see such a dramatic effect between multimerization and LLPS considering the framework’s moderate control over specific multimer formation, e.g. simulations designed to contain tetramers also containing dimers and trimers (Fig. S4). A lack of precise multimer formation somewhat limits our efforts to establish a quantitative relationship between multimerization and LLPS propensity. The trends we observe, however—that higher multimerization states result in greater LLPS propensity—are still valid. At a fixed multimerization state, polymeric multivalency has less impact on LLPS propensity as determined by apparent melting temperature. For any multimer-forming coil it is unknown how many additional coil segments are needed, above a 2-coil protein, to start seeing a significant increase in melting temperature. Additional studies may more clearly show the difference that polymeric versus multimeric multivalency has on these CG CC systems.

In our systems we restricted all CC interactions to interchain interactions. However, intrachain CC interactions will also likely modulate LLPS propensity, as has been seen for IDPs (50). Rana et al. (51) showed by simulation that for proteins with arbitrary numbers of stickers which can undergo both inter- and intrachain interactions, it is the pattern of the stickers that predicts their LLPS propensity. Additionally, it is known that spacers/linkers, and in particular the solvation of linkers, are important in determining LLPS propensity and the properties of resulting condensates (28, 52, 53). We did not explore in our study the effect that different linker size/structure would have on CC protein phase separation. Future studies may investigate how different disordered linkers and CC interaction schemes affect LLPS of CC proteins.

There are several improvements to our system that would enable stronger connection to biologically relevant CC systems. First, while the coil and linker segment lengths were taken as averages of predicted coiled-coils in real systems, the length of individual coil and linker segments can be varied. This would allow us to use the framework to more precisely represent the lengths of real proteins. We have not yet designed the CC LLPS framework to specify parallel vs. antiparallel interaction orientations between coil segments, though these types of orientations are expected in real CC domains (35, 36). We would not expect that orientation-specificity alone would reduce LLPS propensity, but the pattern of coil orientation might be important in dictating protein phase separation behavior. Incorporating parallel and antiparallel coil binding modes is an area of future development for the CC LLPS framework to further its biological relevance for real CC proteins.

Another limitation is the lack of residue-specific interaction terms in the CC LLPS framework. Our current interaction parameters allow us to study the general phenomena of CC-driven LLPS, but adding specific amino acid chemistry into the framework would better capture LLPS of real CC proteins. Recent bespoke CG force fields have been successfully used to study LLPS of IDPs, including those from Dignon et al. (5), Tesei et al. (47), and Joseph et al. (54). We do not expect that these force fields, and particularly the residue-specific interaction parameters, will be directly translatable to our framework because these parameters were refined from data and simulations of IDPs. They will be suitable starting places, however, for us to refine our own residue-level interaction terms specific for coiled-coil proteins.

Finally, real CC domains are thought to often be unfolded as monomers (55–57), and form their distinctive *α*-helical structure upon oligomerization with another CC domain (58, 59). We did not include this behavior in the framework and instead stabilized our coil segments in helical geometry, regardless of multimerization state. This decision was made because we were interested in the phenomena of CC-driven LLPS, not necessarily the mechanisms of CC helical structure formation. Requiring helices to pay a free energy cost of folding before multimerization will affect phase equilibria, and this behavior could be included to further improve the biological relevance of the framework.

Coiled-coil domains as drivers of LLPS represents a new way to think about mechanisms of protein phase separation. The most frequent way to view stickers is as individual residues in intrinsically disordered regions of proteins, despite the flexibility of the stickers-and-spacers framework to be applied to various levels of protein organization (22). This is likely because a majority of proteins identified in biomolecular condensates are intrinsically disordered (60), making IDP components good candidates to study as drivers of LLPS. Our results provide additional evidence that coiled-coil domains could be valid stickers and should be studied in their own right in driving protein phase separation. Our study also justifies studying CC domains as drivers in the phase separation of centrosomal proteins such as spd-5 or pericentrin. Existing data is insufficient to say if CC proteins are the primary domains responsible for LLPS of centrosomal and similar proteins, but these results suggest that future studies to identify driving domains should examine intrinsically disordered and CC domains alike.

## Supplementary Material

Supplement 1

Supplement 2
